# Topographical pathways guide chemical microswimmers

**DOI:** 10.1038/ncomms10598

**Published:** 2016-02-09

**Authors:** Juliane Simmchen, Jaideep Katuri, William E. Uspal, Mihail N. Popescu, Mykola Tasinkevych, Samuel Sánchez

**Affiliations:** 1 Max-Planck-Institut für Intelligente Systeme, Heisenbergstrasse 3, D-70569 Stuttgart, Germany; 2 IV. Institut für Theoretische Physik, Universität Stuttgart, Pfaffenwaldring 57, D-70569 Stuttgart, Germany; 3 Institut de Bioenginyeria de Catalunya (IBEC), Baldiri I Reixac 10-12, 08028 Barcelona, Spain; 4 Institució Catalana de Recerca i Estudis Avancats (ICREA), Pg. Lluís Companys 23, 08010 Barcelona, Spain

## Abstract

Achieving control over the directionality of active colloids is essential for their use in practical applications such as cargo carriers in microfluidic devices. So far, guidance of spherical Janus colloids was mainly realized using specially engineered magnetic multilayer coatings combined with external magnetic fields. Here we demonstrate that step-like submicrometre topographical features can be used as reliable docking and guiding platforms for chemically active spherical Janus colloids. For various topographic features (stripes, squares or circular posts), docking of the colloid at the feature edge is robust and reliable. Furthermore, the colloids move along the edges for significantly long times, which systematically increase with fuel concentration. The observed phenomenology is qualitatively captured by a simple continuum model of self-diffusiophoresis near confining boundaries, indicating that the chemical activity and associated hydrodynamic interactions with the nearby topography are the main physical ingredients behind the observed behaviour.

## Abstract (web summary)

Self-propelled colloids can be used as cargo carriers, but it is challenging to control their motion without external fields. Here, Simmchen *et al*. use submicron patterns on a solid substrate to effectively confine the motion of the chemically active Janus microswimmers along the edges of the patterns.

## Graphical abstract

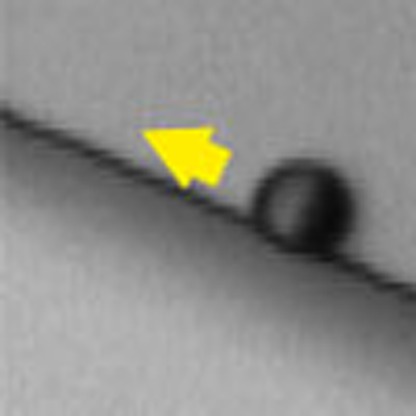

Catalytically active micrometre-sized objects can self-propel by various mechanisms including bubble ejection, diffusio- and electrophoresis, when parts of their surface catalyse a chemical reaction in a surrounding liquid. In future, such chemically active micromotors may serve as autonomous carriers working within microfluidic devices to fulfill complex tasks^1^,^2^,^3^. However, to achieve this goal, it is essential to gain robust control over the directionality of particle motion. Although it has been more than a decade since motile chemically active colloids were first reported^4^,^5^,^6^,^7^, this remains a challenging issue, in particular for the case of spherical particles.

Two main methods of guidance have been so far employed with varying degrees of success. The first one uses controlled spatial gradients of ‘fuel' concentration. This approach suffers, however, from severe difficulties in creating and maintaining chemical gradients, and the spatial precision of guidance remains rather poor^8^,^9^,^10^,^11^,^12^. The second approach relies on the use of external magnetic fields in combination with particles with suitably designed magnetic coatings or inclusions^5^,^13^. This proved to be a very precise guidance mechanism, which could be employed straightforwardly for the case of tubular particles^14^, but difficult to extend to the case of spherical colloids, where it requires sophisticated engineering of multilayer magnetic coatings^7^,^15^,^16^,^17^. In addition, individualized guidance of specific particles is difficult to achieve without complicated external apparatus and feedback loops^18^. The advantages of autonomous operation are thereby significantly hindered.

Although these methods are quite general in their applicability, we note here that the synthetic micromotors are, in general, density mismatched with the suspending medium and therefore tend to sediment and move near surfaces. Furthermore, even in situations in which sedimentation can be neglected (for example, in the case of neutrally buoyant swimmers), the presence of confining surfaces has profound consequences on swimming trajectories, as discussed below. Theoretical studies have shown that long-range hydrodynamic interactions (HIs) between microswimmers and nearby surfaces^19^,^20^ can give rise to trapping at the walls or circular motion. Moreover, a theoretical study of a model active Janus colloid moving near a planar inert wall has revealed complex behaviour, including novel sliding and hovering steady states^21^. Experimentally, wall-bounded motion of active Janus particles was evidenced in the study by Bechinger *et al*.^22^, whereas capture into orbital trajectories of active bimetallic rods by large spherical beads or of Janus colloids in colloidal crystals^23^ has been reported in recent times. Capture of microswimmers by spherical obstacles via HIs has been modelled theoretically by Lauga *et al*.^24^.

This intrinsic tendency of the active swimmers to operate near bounding surfaces motivated us to examine whether it can be further exploited to achieve directional guidance of chemically active microswimmers by endowing the wall with small height step-like topographical features, as shown in Fig. 1, which the particles can eventually exploit as pathways. Recently, Palacci *et al*.^25^ have shown that shallow rectangular grooves can efficiently guide photocatalytic hematite swimmers that have size comparable with the width of the groove. Because of the strong lateral confinement, it is hard to discriminate between the different physical contributions that lead to particle guidance. Here we use a much less restrictive geometry—a shallow topographical step—and it is *a priori* not clear whether a self-phoretic swimmer can follow such features. We report experimental evidence that Janus microswimmers can follow step-like topographical features that are only a fraction of the particle radius in height. This is, in some sense, similar to the strategy employed in natural systems well below the microscale: within cells, protein motors such as myosin, kinesin and dynein use binding to microtubules to switch to directional motion^26^,^27^. The guidance of microswimmers through patterned device topography that we propose and demonstrate in this study may pave the way for new methods of self-propeller motion control based on patterned walls.

## Results

### Dynamics of Janus microswimmers at a planar wall

**Figure 1:**
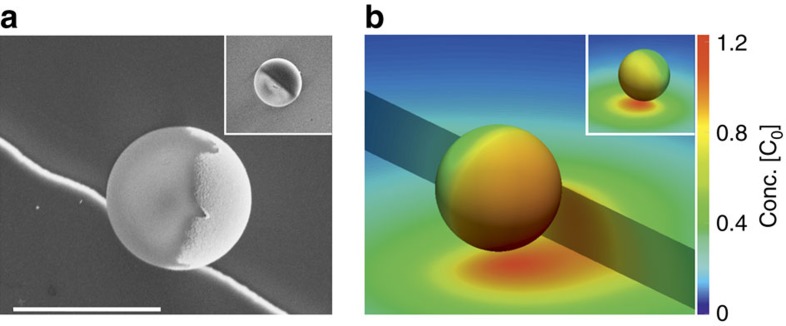
Janus microswimmers near submicrometre steps. (**a**) Top view: scanning electron microscopy (SEM) image of a spherical Janus motor on a silicon substrate with a silicon step. The lighter part of the Janus particle corresponds to Pt, while the grey part is the SiO_2_; scale bar, 2 μm. (**b**) Colour-coded steady-state distribution *c*(**r**) of reaction products around a half covered Janus particle at an inert wall and a step with height *h*_step_=*R*, where *R* is the particle radius. The colour map shows *c*(**r**) at the surfaces of the particle and substrate, and is represented in units of *c*_0_ defined in Methods. The insets show the particles on a flat surface.

Janus particles are fabricated by vapour deposition of a thin layer of Pt (7 nm) on SiO_2_ particles (diameters of ∼2 and 5 μm). For details on the fabrication, see Methods section. Scanning electron microscopy images of a Janus particle at the step edge are shown in Fig. 1a, whereas Fig. 1b illustrates numerically calculated steady-state distributions of the reaction products around a model half Pt-covered Janus sphere near a step.

**Figure 2:**
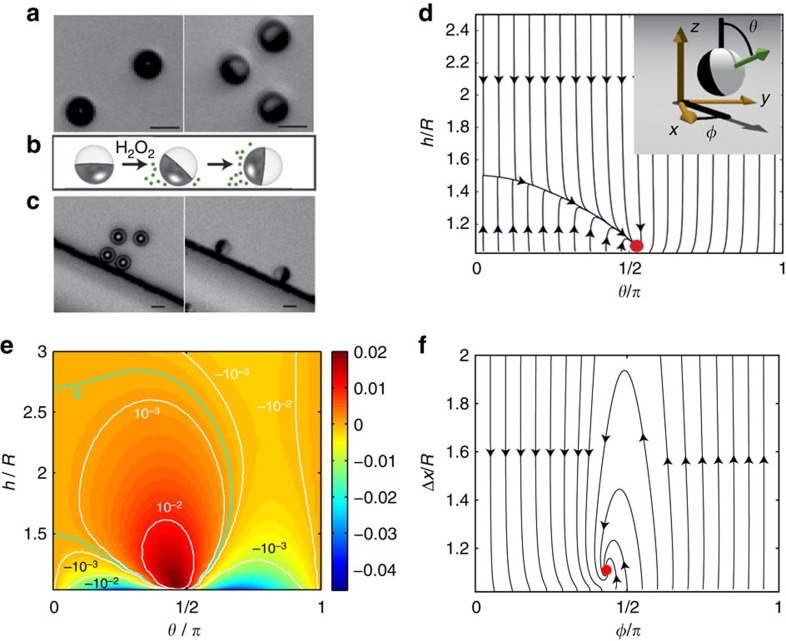
Behaviour of Janus particles near planar surfaces. (**a**) Particles that sedimented to the bottom surface in a water suspension (inactive system) tend to align with their Pt caps facing downwards, which is more pronounced for larger particles. The Pt caps (*ρ*=21.45 g cm^−3^), which are much denser than the silica parts of the particles, render them bottom heavy (in the image one sees transparent SiO_2_ on top of heavily absorbing Pt). However, on addition of H_2_O_2_ (active system), the particles reorient their symmetry axis parallel to the bottom surface and can be seen as half-covered circles in the right micrograph in **a**, where dark semi-spheres correspond to the Pt cap and the SiO_2_ parts that do not absorb the light appear lighter. (**b**) Schematic of a particle rotating from bottom-down configuration on peroxide addition. (**c**) Micrograph of Janus colloids (*R*=2.5 μm) in the vicinity of the step with height *h*_step_=800 nm. In the absence of H_2_O_2_ (left image), the step (seen as a black line) has no influence on the orientation of the particles, (their caps are facing downwards, same as far from the step). On addition of fuel (right image), the particles orient with their symmetry axis parallel to both the bottom surface and the step. Scale bars, 5 μm (**a**,**c**). (**d**) The phase portrait for a bottom-heavy (see Supplementary Note 1) particle with *R*=2.5 μm at an infinite planar wall oriented with its normal parallel to the direction of gravity. The phase portrait is calculated at *b*_inert_/*b*_cap_=0.3 and *b*_w_/*b*_cap_=−0.2, where *b*_inert_ and *b*_cap_ are the surface mobilities (see Methods for details) at the inert and catalytic faces of the particle, respectively, and *b*_w_ is the surface mobility at the wall (the phase portrait for *R*=1.0 μm is shown in Supplementary Fig. 2). The phase portrait indicates that a particle will rotate to its steady-state orientation *θ*=*θ*_eq_≈90° for all initial conditions. The inset represents a schematic diagram of the system: a Janus sphere of radius *R* is placed at distance *h* above an inert wall; *θ* describes the orientation of the particle's cap with respect to the wall normal. Δ*x* is the step particle distance and *φ* is the cap orientation with respect to the step normal. (**e**) The rate of rotation 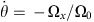 of a particle with *R*=2.5 μm above a planar substrate, including contributions from activity, gravity and chemiosmotic flows on the substrate. This function is the sum of Fig. 3a,e,f. (**f**) Phase portrait similar to the one in **d** but in absence of gravity. All other parameters are as in **d**; this portrait is supposed to capture qualitatively the effect of the vertical step wall.

Initially, the particles are introduced to the system with no H_2_O_2_ present and due to their weight they sediment near the bottom surface. After sedimentation, we find the particles uniformly distributed over the substrate and most of the 5-μm particles have their much denser (compared with the SiO_2_ cores) Pt caps oriented downwards (Fig. 2a left), while smaller particles have a wider distribution of orientations. The particles are seen in the same focal plane of the microscope, which indicates that they are at similar vertical distances from the substrate. On addition of H_2_O_2_ to the system, we observe that the Pt caps of the microswimmers are oriented parallel to the substrate plane (see Fig. 2a right and Fig. 2b, and for the definition of the geometrical parameters see Supplementary Fig. 1). Following this reorientation, the microswimmers start moving parallel to the substrate in the direction away from the catalytic caps. In Fig. 2c we show snapshots from an optical microscopy video recording of Janus microswimmers in the vicinity of a step with *h*_step_=800 nm. Similar to the case depicted in Fig. 2a (left), in the absence of H_2_O_2_ the particles are oriented cap down. After addition of hydrogen peroxide, the particle caps turn away from the substrate and the particles start moving in random directions until some of them encounter a step; if the step is sufficiently tall (depending on the particle size) the particles stop, reorient and continue self-propelling along it. These observations confirm our hypothesis that the presence of a side step near the active microswimmers, even if small compared with the particle radius, has an influence on their orientation.

We show that this behaviour (alignment with both the wall and the step) is captured by a simple model of neutral self-diffusiophoresis (see Methods section for details of the model), in which we assume that the activity of the Janus particle is captured by the release of a neutral solute (O_2_ molecules) at a constant rate from its catalytic cap^28^. The resulting anisotropic solute distribution around the particle drives a surface flow in a thin layer surrounding the particle, leading to its directed motion^28^,^29^. The catalytically active particle has several types of interaction with a nearby impermeable wall. The particle drives long-range flows in the suspending solution. These flows are reflected from the wall, coupling back to the particle (HI). Second, the particle's self-generated solute gradient is modified by the presence of the wall. The wall-induced modification of the solute concentration field can contribute to translation and rotation of the particle (‘phoretic interaction'). In particular, when the solute interacts more weakly with the inert region of the particle than with the catalytic cap and both interactions are repulsive (see Methods for details), the confinement and accumulation of solute near the substrate tends to drive rotation of the cap away from the substrate. On the other hand, the bottom heaviness of the particle, along with the HI of the particle with the substrate, tends to drive the rotation of the cap towards the substrate. Finally, the inhomogeneous solute distribution along the wall induces a solute gradient-driven ‘chemiosmotic' flow along the substrate. For repulsive solute–substrate interactions, this surface slip velocity is directed quasi-radially inward towards the particle, driving a particle-uplifting flow in the suspending solution and causing the particle cap to rotate away from the substrate. For attractive solute–substrate interactions, the opposite directions of flows apply. Numerical analysis of this model system shows that depending on the relative strengths of these interactions (that is, the parameters characterizing the surface chemistry of the particle and the wall), the various contributions to rotation discussed above may balance at a steady height *h*_eq_ and orientation *θ*_eq_≈90°, and that this steady state is robust and stable against perturbations in height and orientation. The particle cap orientation would therefore evolve to *θ*_eq_≈90° (that is, the symmetry axis almost parallel to the substrate) from nearly all initial orientations, including a cap-down one. In Fig. 2d, a phase portrait shows the dynamical evolution of particle height and orientation, and the colour-coded rate of rotation 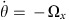 is depicted in Fig. 2e. The steady state (red dot) clearly has a large basin of attraction. We note that our numerical calculations were carried out for *h*/*R*⩾1.02. Therefore, some trajectories in the region of the cap up (*θ*=180°) orientation encounter a numerical cutoff. However, based on the structure of the phase portrait, we expect such trajectories to roll towards *θ*≈90° after a close encounter with the wall.

If a particle as above would encounter now a second vertical side wall, numerical simulations for the same interaction parameters show (see Fig. 2f) that for this wall, for which gravity now plays no role, a similar sliding along the wall attractor emerges with *φ*_eq_≈90°, that is, with the particle oriented with its axis almost parallel to the vertical wall. The combination of the two sliding states thus aligns the axis of the particle along the edge formed by the two walls. It is noteworthy that although this second fixed point appears to have a smaller basin of attraction, it should capture the whole *φ*≤*π*/2 range. A particle on a trajectory that ‘crashes' into the vertical wall would diffuse along the wall until it reaches the basin of attraction in the vicinity of *φ*_eq_≈90°. Although the argument is developed for the superposition of two infinite planar walls, we expect that similar features may occur for a vertical step with finite height. The above results will also hold for particles with the catalytic cap less dense than Pt. It is easy to see that in this case, while keeping all the other parameters of the system, such as geometry of the cap, activity and so on, fixed, a sliding fixed point along the bottom wall will also emerge. Moreover, we have checked via numerical simulations (results not shown) that the corresponding height and the orientation will lie between the values corresponding to the fixed points shown in Fig. 2d,f and thus the corresponding orientation will remain close to 90°.

**Figure 3:**
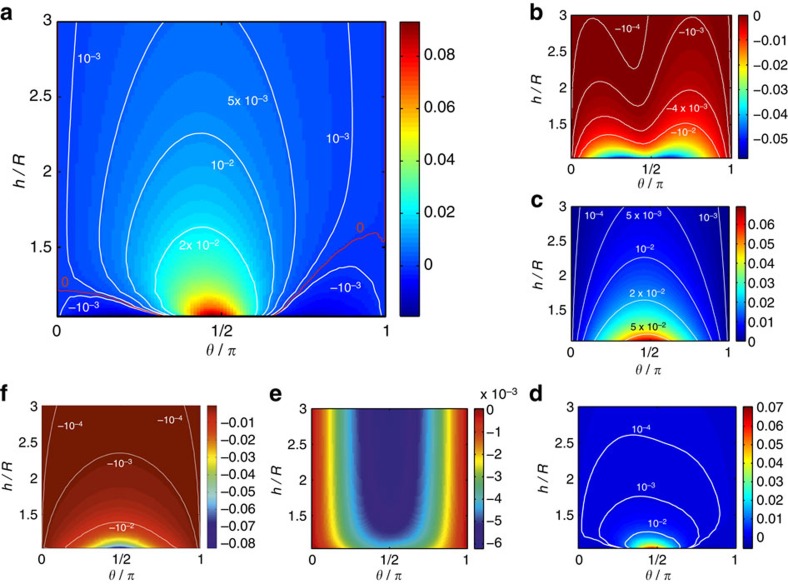
Various contributions to particle angular velocity Ω_*x*_. (**a**) Contribution from self diffusiophoresis 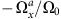 (see Supplementary Note 2 and equation (2) as a function of height *h*/*R* and orientation *θ* for half-covered Janus microswimmer and unequal surface mobilities *b*_inert_/*b*_cap_=0.3. Throughout, white curves correspond to constant values of 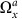. Note that, by definition, panel **a** is the sum of panels **b**,**c**, and **d**. (**b**), Contribution 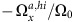 obtained by using the free space number density of solute distribution *c*^*fs*^(**r**) around the particle, i.e., neglecting the influence of the wall on the number density of solute, but including the influence of the wall on the hydrodynamic flow. (**c**) Contribution 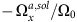 obtained by using the free space hydrodynamics stress tensor 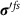 in the dual Stokes problems employed in the reciprocal theorem, i.e., neglecting the effect of the wall on the hydrodynamics, but including the chemical effect. (**d**) Contribution 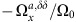 due to higher order coupling between the two effects. (**e**) Contribution to rate of rotation from the bottom-heaviness of the particle. (**f**) Chemio-osmotic contribution 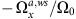 due to the activity-induced phoretic slip at the wall calculated at *b*_*w*_/*b*_cap_=−0.2.

Within our model, we can isolate and quantify the various wall-induced contributions to particle motion discussed above. The mathematical details of the decomposition are given in Supplementary Note 2. In Fig. 3b, we show the contribution to the rate of rotation 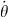 of the particle from HI with the wall as a function of particle height and orientation. HIs always rotate the particle cap towards the wall. Therefore, for the particular combination of parameters used in this work, HIs cannot by themselve*s* produce a steady orientation *θ*_eq_≈90°. On the other hand, phoretic interactions always rotate the cap away from the wall, as described above (Fig. 3c). Therefore, the interplay of hydrodynamic and phoretic interactions can produce a curve with 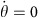 in the region of *θ*_eq_≈90° (Fig. 3a). Moreover, the contributions of bottom heaviness (Fig. 3e) and chemiosmotic flow on the wall (Fig. 3f) to the angular velocity are comparable in magnitude with the contributions from HI and phoretic interactions. Therefore, for the parameters used in this work, all of these effects are important in determining the emergence and location of a ‘sliding state' attractor. The surface chemistry parameters were chosen as providing the best fit to the experimental observations of the two sliding states (above a substrate and along a side wall).

As noted above, our model includes several types of interaction of the particle with the wall. However, many theoretical^24^ and experimental^22^,^23^ studies have sought to characterize the interaction of active particles and solid boundaries strictly in terms of effective HIs. It is therefore interesting to compare our full model against the best fit results from effective HI models. We consider two such approaches, the details of which are in Supplementary Note 3. Briefly, in the first approach, we use the classical ‘squirmer' model and specify *a priori* the amplitude of the first two squirming modes. Higher-order modes are taken to have zero amplitude. In the second approach, we consider the ‘effective squirmer' obtained within our model by neglecting phoretic and chemiosmotic effects. The ‘effective squirmer' approach intrinsically covers a broad range of squirming mode amplitudes. In Table 1, we show that the results of the full model match the experimental observations significantly better than the best results of the two HI-only approaches.

### Dynamics of Janus microswimmers at a rectangular step

We designed a system with microfabricated three-dimensional structures by patterning of photoresist through a circular or square mask, followed by e-beam deposition of the required material (Si or SiO_2_ in our case), and the removal of the developed photoresist resulting in desired structures (for detailed information, see Methods). Depending on the use of positive or negative photoresist, we obtain patterns with posts or wells of different shapes (see Supplementary Fig. 5). The height of the features patterned on a substrate is tunable in a wide range; in this study, we have tested step heights *h*_step_ between 100 and 1,000 nm.

### Characterization of particle trajectories approaching a step

**Figure 4:**
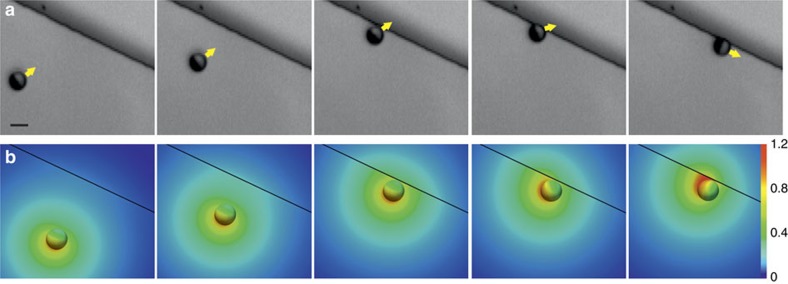
Effect of 800 nm height step on the dynamics of a Janus microswimmer. (**a**) An active Janus particle approaching a step; after direct contact with the step, it reorients until its propulsion axis is parallel to the step. *R*=2.5 μm, *h*_step_=800 nm, 2.5%vol. H_2_O_2_. (**b**) Numerically calculated steady-state distribution *c*(**r**) of reaction products around half catalyst-covered Janus sphere as a function of the step distance and the cap orientation with respect to the step. The colour map shows *c*(**r**) at the surfaces of the particle and substrate; *c*(**r**) is in units of *c*_0_ (see Methods). Scale bar, 5 μm.

Figure 4a shows snapshots of a typical trajectory of a microswimmer moving towards a step at almost perpendicular direction. Once the particle hits the step (Fig. 4a third panel) it starts reorienting its axis (Fig. 4a fourth panel) towards the direction along the step (Fig. 4a fifth panel). We observe that in most cases the complete process of reorientation takes <10 s, independent of the initial angle at which the particle approaches the step. Within the resolution of our experimental equipment, we do not observe any systematic deflection in the trajectory of the particle in the vicinity of the steps. Therefore, we conclude that if any long-range effective interaction exists between the particles and the steps, it must be very weak. This observation is reproduced by our numerical model: we calculate that the effects of a wall on the velocity of a particle are negligible when the particle is more than three radii away from the wall (Supplementary Fig. 4 and Supplementary Note 4). We attribute the suppression of the motion of the particles in the direction normal to the step upon collision to purely steric interactions.

In Fig. 4b we present the distribution of the reaction products around a microswimmer calculated numerically for particle positions and orientations approximately corresponding to those shown in the experimental micrographs in Fig. 4a. When the particle is far away from the step (Fig. 4b first and second panels), approaching it in a head-on direction, the generated concentration field confirms the expected mirror symmetry with respect to the plane defined by the motion axis and normal to the substrate. As the result of this symmetry, there are no activity-induced rotations and the particle stays on its head-on track (up to Brownian rotational diffusion) towards the step. However, closer to the step, a head-on collision becomes unstable to small fluctuations of the propulsion axis, as any such fluctuation gets amplified by the buildup of asymmetric product distribution in the region between one side of the particle and the step (Fig. 4b fourth panel). This eventually leads to the reorientation of the motion axis parallel to the step (Fig. 4b fifth panel).

### Effects of the step height on the capture efficiency

**Figure 5:**
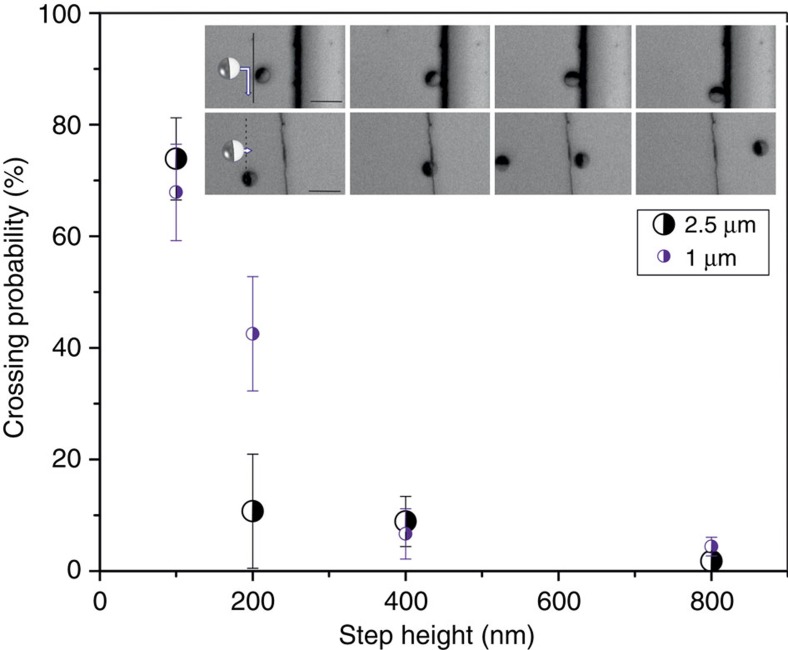
Submicrometre steps as rectifiers of active particles' trajectories. A summary of the crossing behaviour of Janus SiO_2_ microswimmers of different sizes at several values of *h*_step_; the error bars are s.e.m. Inset: a sequence of micrographs showing a Janus particle with *R*=2.5 μm approaching a step with *h*_step_=800 nm, reorienting and then moving parallel to it (upper row). Micrograph sequence of a Janus particle, 2.5 μm, passing over a step, *h*_step_=100 nm (lower row). Scale bars, 10 μm (all).

We observe that submicrometre steps are able to capture and guide particles as shown in Fig. 5 (insets). To evaluate the minimum height 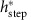 that can still influence the trajectory of the particles, we fabricated a set of patterns with *h*_step_ varying in a range from 100 to 1,000 nm. The results for the two different particle sizes show that 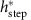 decreases as the particles size increases. In Fig. 5 we summarize the responses of *R*=1 μm and *R*=2.5 μm active particles to variation of the step height. Both types of particles can swim over the step of 100 nm height. For *R*=2.5 μm particles, steps of height 200 nm already ensure ∼90% docking of particles upon collision with the step, while a significant fraction of the *R*=1 μm particles managed to pass over the 200-nm-high step, and 400-nm-high steps were required for efficient docking. From Fig. 5 we infer that 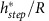 is smaller for larger particles.

Having estimated the threshold values 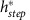 for particle trapping, we now select steps of sufficient height to ensure full trapping upon collisions. Therefore, all the following experiments were carried out on 800 nm features, for which both 2.5 and 1 μm particles follow the step upon collision.

### Guidance of microswimmers by low-height topographic steps

**Figure 6:**
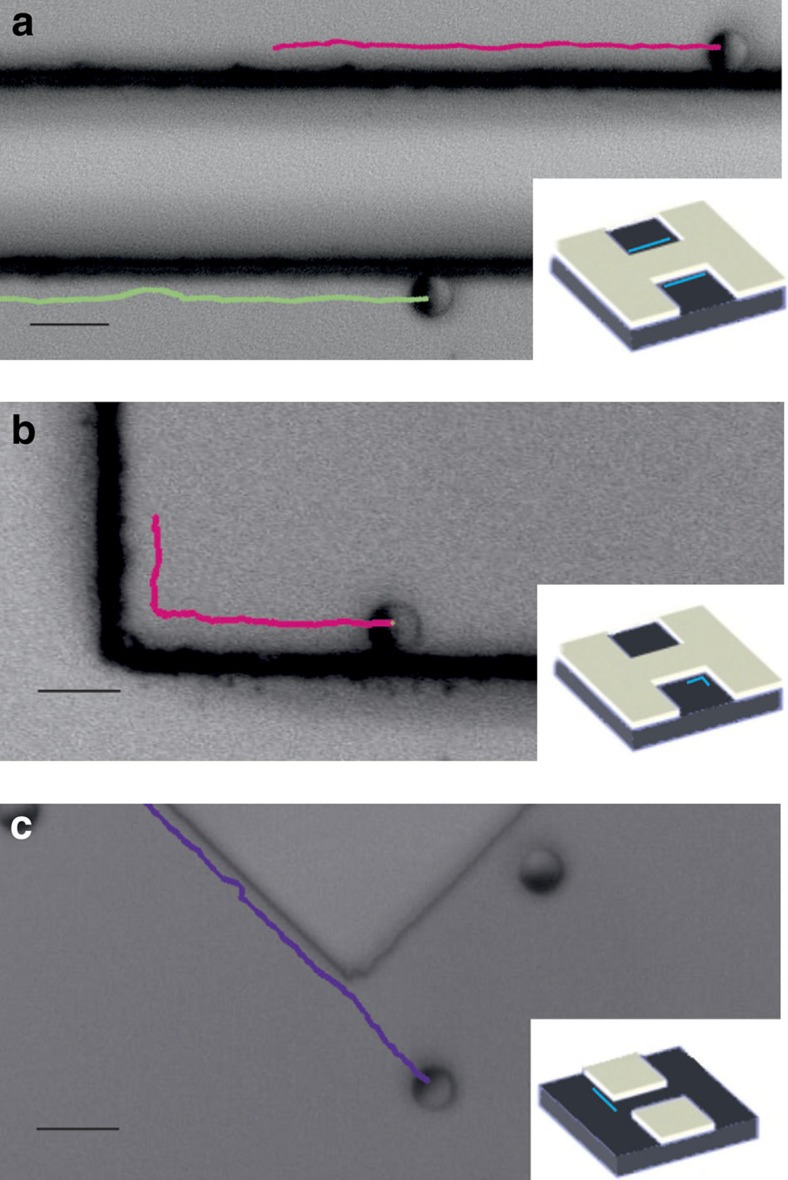
Guidance of Janus microswimmers by step features. (**a**) A micrograph showing trajectories of two *R*=2.5 μm Janus particles following a straight step. (**b**) A Janus particle tracked while manoeuvring around a 90° corner. (**c**) A Janus particle unable to follow a reflex angle of 270°. The insets show schematically the structures of wells **a**,**b** and posts **c**. The blue lines on the insets schematically indicate the position of Janus particles in actual experiments. Scale bars, 10 μm.

The step-like topography and particle alignment along step edges can be used to guide microswimmers, as it is shown in Fig. 6a. This corresponds to particles in a well structure with straight steps. Upon collisions, the particles align along the steps and follow them (Fig. 6a and Supplementary Movie 1). In the same well-like structure, particles eventually encounter a corner and after spending some time adjusting their orientation can manoeuvre around the corner (Fig. 6b and Supplementary Movie 2). In the case of a post-like structure as displayed in Fig. 6c, particles also follow straight features but fail to reorient and manoeuvre around the 270° corner (see also Supplementary Movie 3). These findings suggest that certain critical value must exist for reflex angles between features above which guidance along the step is lost.

**Figure 7:**
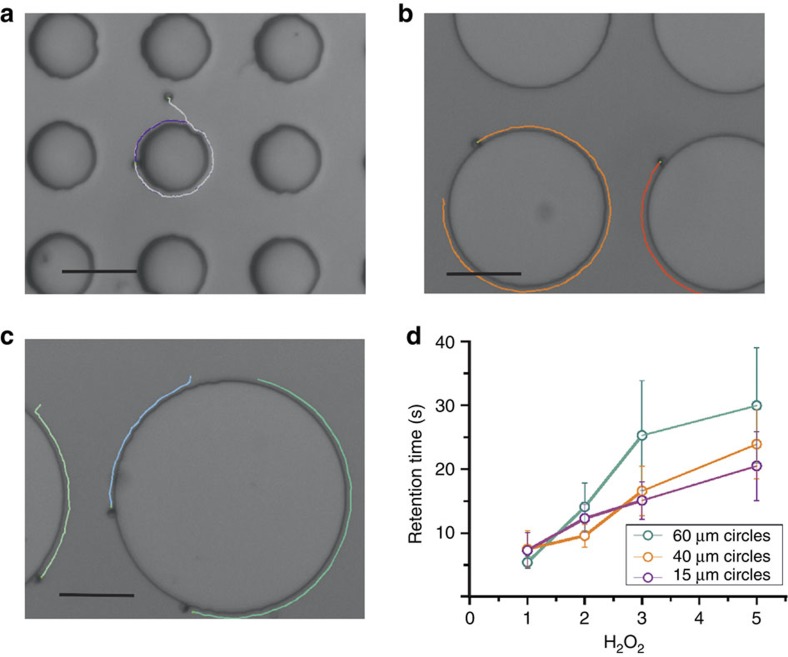
Trapping and guiding active particles by circular posts. (**a**–**c**) Optical snapshots of *R*=1 μm active particles moving for 12 s around circular posts of 15, 40 and 60 μm diameter *d*, respectively. Scale bar, 20 μm. (**d**) Average retention time as a function of peroxide concentration for *R*=1 μm. The average is determined for 15–20 trajectories per data point; the error bars are standard errors of the mean.

Active Janus microswimmers also follow circular trajectories around circular posts as shown in Fig. 7, which requires constant reorientation of the axis. In Fig. 7a–c, paths are shown where particles with *R*=1 μm circle around posts with the diameter of 15, 40 and 60 μm, respectively, for more than 12 s (see Supplementary Movies 4–6). Supplementary Fig. 3 and Supplementary Movie 7 show an additional example of cycling motion with long retention times. We find that the retention time of microswimmers at the circular posts increases with increasing peroxide concentration, as displayed in Fig. 7d. At 1%, few particles completely circle around a whole post and, in most cases, the microswimmers detach from the post before a complete revolution (at lower peroxide concentrations, the particles hardly move and get easily stuck at the steps; thus, measurements were not considered). At 2% H_2_O_2_, the path length along the posts is increased and likewise in 3 and 5% H_2_O_2_, where many particles circle around posts multiple times. At even higher concentrations of H_2_O_2_, we observe vigorous formation of oxygen bubbles and occurrence of convective flows, and thus no reliable measurements could be performed above 5% H_2_O_2_.

As the retention time increases with the concentration of H_2_O_2_, we conclude that it is the activity of the microswimmers that is directly responsible for the effective particle attraction to the posts and for the occurrence of the sliding attractor: it is the net result of the particle-step HI and confinement-induced modification of the distribution of the oxygen concentration. The strength of both effects depends on the fuel concentration: increased fuel concentration leads to a higher production rate of solute (that is, stronger phoretic and chemiosmotic interactions) and a higher self-propulsion velocity (that is, stronger HIs). The finite retention time is set by the competition between activity-induced effective attraction to the post side walls and rotational diffusion. Increased fuel concentration increases the strength of the first factor without affecting the second one and therefore increases the retention time. The robustness of the sliding state attractor is further discussed in Supplementary Note 5.

## Discussion

We report experimental results showing the dynamics of chemically active Janus microswimmers at geometrically patterned substrates and a qualitative interpretation in terms of a minimal continuum model of self-diffusiophoresis of chemically active colloids. Employing a lithography-based method to fabricate submicrometre topographic features in the form of rectangular stripes, square posts, cylindrical posts or square wells on glass surface or silicon wafer, we demonstrate that the motion of chemically active Janus microswimmers can be restricted to proceed along these small height patterns for significant time intervals. Furthermore, the motion along the circumference of cylindrical posts reveals that the retention time increases with increasing H_2_O_2_ concentration. This allows us to unequivocally identify the particle's chemical activity, which modulates the distribution of the phoretic slip at the particle surface and thus the HI with the nearby topography, as playing a dominant role in the observed phenomenology.

We also show that a minimal continuum model of self-diffusiophoresis captures the qualitative features of the experimental observations if one accounts for the difference in material properties of the two parts of the colloid and for chemiosmotic flows induced at the wall. This latter aspect highlights the need for models that explicitly include chemical activity, without which a no-slip boundary condition would apply at the wall. The model exploited here allows us to understand the emergence of states of motion along the edges as a simultaneous attraction to two fixed-point attractors corresponding to steady sliding states along the bottom wall and along the vertical wall of the step.

The microstructuring method presented here avoids the use of any external fields and relies solely on the intrinsic properties of the system to control particle motion. The phenomenology reported here is, in some sense, a mesoscale analogue of the binding of motor proteins to microtubules, to switch to directional motion. However, in distinction to biological nanomotors, the Janus microswimmers bypass the binding and rather elegantly exploit an effective attraction that stems from the feedback between geometric confinement, and chemical and hydrodynamic activity. The results presented here open the possibility of robust guidance of particles along complex paths via minimal surface modifications, that is, by sculpting a pattern with the edge in the desired shape. This may have significant implications in designing new applications based on artificial swimmers. Finally, we consider that these findings will allow further developments by employing smart, chemically patterned walls, where features of the nearby surfaces (and thus the guiding of the microswimmers) can be turned on and off.

## Methods

### Sample preparation

Janus particles were obtained by drop casting of a suspension of spherical silica colloids (diameter of 2 or 5 μm, Sigma Aldrich) on an oxygen-plasma cleaned glass slide followed by slow evaporation of the solvent and subsequent placement in an e-beam system. High vacuum was applied and subsequently a monolayer of 7 nm Pt was evaporated, to guarantee catalytic properties. To release particles from the glass slides into deionized water, short ultrasound pulses were sufficient.

Photoresist patterns were prepared on 24 mm square glass slides or on silicon wafers. In case of positive photoresist, AR-P 3,510 was spin coated onto the cleaned substrate at 3,500 r.p.m. for 35 s, followed by a soft bake using a hotplate at 90 °C for 3 min and exposure to ultraviolet light with a Mask Aligner (400 nm) for 2 s. Patterns were developed in a 1:1 AR300-35:H_2_O solution. In case of negative photoresist, a layer of adhesion promoter TI prime was spin coated on the substrate during 20 s at 3,500 r.p.m. After 2 min of drying at 120 °C, the negative photoresist was coated employing a programme of 35 s spinning at 4,500 r.p.m., followed by 5 min baking at 90°. The exposure was carried out with a Mask aligner for 2 s followed by 2 min on the hotplate at 120 °C. Finally, an additional exposure to 2 s ultraviolet light is applied and the patterns were developed in pure AZ726MIF. The steps were obtained by e-beam deposition of the desired material (SiO_2_, Si) in the desired thickness. By dissolving the photoresist layer in acetone the pattern structures of the substrate are exposed; the whole process is illustrated in Supplementary Fig. 5.

Before experiment, the patterned substrates were cleaned by oxygen plasma. Experiments were performed directly on the substrates by adding equal volumes of particles in deionized water and diluted peroxide solutions. Videos were recorded with a Leica DFC 300G camera mounted to a Leica upright microscope at ∼30 fps. Evaluation and tracking was performed using Fiji analysis software.

### Tracking

Accurate tracking of Janus particles was performed automatically by a specially developed script in Python 2.7 using the OpenCV library. The position of the Janus swimmers at every frame is found by extracting the background, which erases the static posts from the image, leaving only the moving particles.

### Theoretical modelling

We model particle motion within a continuum, neutral self-diffusiophoretic framework. A particle emits solute at a constant rate from its catalytic cap. The number density *c*(**r**) of solute is quasi-static, where **r** is a position in the fluid. The solute field is governed by the Laplace equation ∇^2^*c*=0 and obeys the boundary conditions −*D*∇*c*·**n**=*κ* on the catalytic cap and −*D*∇*c*·**n**=0 on the inert face of the particle and the substrate, where *κ* is the rate of emission (uniform over the cap), *D* is the diffusion coefficient of oxygen and **n** is the local surface normal. Our model neglects the details of the catalytic reaction, which might involve the transport of charged intermediates^28^,^29^. Nevertheless, we expect this model to capture the gross effects of both near-wall confinement of the solute field and HI with nearby walls. The surface gradient of solute drives a surface flow (‘slip velocity') **v**_s_ in a thin fluid layer surrounding the particle surface **v**_s_(**r**)=−*b*_s_(**r**)∇_||_*c*, where 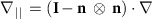. **I** is the 3 × 3 unit matrix.

The coefficient *b*_s_(**r**) of the slip velocity, the so-called ‘surface mobility', is determined by the molecular interaction potential between the solute and the particle surface^30^. We allow *b*_s_(**r**) to differ between the inert and catalytic regions, but assume it is uniform in each region, that is, take *b*_s_=*b*_inert_ or *b*_s_=*b*_cap_. In addition, when we consider the effect of chemiosmotic flow on the substrate, we calculate a wall slip velocity **v**_w_(**r**_s_)=−*b*_w_∇_||_c, where *b*_w_ is a constant. We always take the interaction between the solute and particle surface to be repulsive, that is, *b*_s_<0, so that the model is consistent with the observed motion of particles away from their caps.

The velocity **u**(**r**) in the fluid is governed by the Stokes equation −∇*p*+*η*∇^2^**u**=0 and the incompressibility condition ∇·**u**=0, where *p*(**r**) is the fluid pressure and *η* is the dynamic viscosity of the solution. The velocity obeys the boundary conditions **u**=**v**_w_(**r**) on the substrate and **u**(**r**)=**U**^*a*^+**Ω**^*a*^ × (**r**−**r**_O_)+**v**_s_(**r**) on the particle surface, where **r**_O_ is the position of the particle centre and **U**^*a*^ and **Ω**^*a*^ are the contributions of activity to the translational and rotational velocities of the particle, respectively. To obtain **U**^*a*^ and **Ω**^*a*^ for a given position and orientation of the particle, we first solve for *c*(**r**) numerically, using the boundary element method^31^. The slip velocities **v**_s_ and **v**_w_ are then calculated from *c*(**r**). Inserting the slip velocities in the boundary conditions and requiring that the particle is force and torque free, we solve the Stokes equation numerically via the boundary element method, to obtain **U**^*a*^ and Ω^*a*^ in terms of characteristic velocity scales 
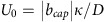
 and 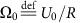. In addition, *c*(**r**) is calculated in terms of a characteristic concentration 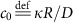.

When we include the effects of gravity, we adopt the geometrical model of Campbell and Ebbens^32^, taking the Janus particle as having a platinum cap that smoothly varies in thickness between a maximum of 7 nm at the pole and zero thickness at the particle equator. The gravitational contributions to particle velocity, **U**^*g*^ and **Ω**^*g*^, are calculated using standard methods (see Supplementary Note 1).

We obtain complete particle trajectories by numerically integrating **U**=**U**^*a*^+**U**^*g*^ and **Ω=Ω**^*a*^+**Ω**^*g*^. Further details of the numerical method are given in ref. 21. We note that the assumption that the solute field is quasi-static is valid in the limit of small Peclet number Pe=*U*_0_*R*/*D*. We have neglected the inertia of the fluid, which is valid for small Reynolds number Re=*ρU*_0_*R*/*η*, where *ρ* is the mass density of the solution. These dimensionless numbers are Pe≈4 × 10^−3^ and Re≈10^−5^ for a 5-μm-diameter catalytic Janus particle that swims at 6 μm s^−1^ (ref. 33).

## Additional information

**How to cite this article:** Simmchen, J. *et al*. Topographical pathways guide chemical microswimmers. *Nat. Commun.* 7:10598 doi: 10.1038/ncomms10598 (2016).

## Supplementary Material

Supplementary Information Supplementary Figures 1-5, Supplementary Tables 1-2, Supplementary Notes 1-5 and Supplementary References

Supplementary Movie 1 Two R=2.5 μm microswimmers in 5 vol/vol% H2O2 following a straight step (180 degrees) and one moving nearly parallel to the step for some distance.

Supplementary Movie 2 R=2.5 μm microswimmer in 5 vol/vol% H2O2 maneuvering a 90° angle.

Supplementary Movie 3 R=2.5 μm microswimmer in 5 vol/vol% H2O2 detaching from the step feature when it encounters a 270° angle.

Supplementary Movie 4 R=1 μm microswimmer in 5 vol/vol% H2O2 following circular steps with a diameter of 15 μm.

Supplementary Movie 5 R=1 μm microswimmer in 5 vol/vol l% H2O2 following circular steps with a diameter of 40 μm.

Supplementary Movie 6 R=1 μm microswimmer in 5 vol/vol % H2O2 following circular steps with a diameter of 60 μm.

Supplementary Movie 7 R=2.5 μm microswimmer following circular steps with a diameter of approx. 15 μm.

## Figures and Tables

**Table 1:** Comparison of full model with two hydrodynamics-only models.

**Model**	**Best fit parameters**	***h***_**eq**_**/*****R*****, no gravity**	***θ***_**eq**_**, no gravity**	***h***_**eq**_**/*****R*****, with gravity**	***θ***_**eq**_**, with gravity**
Full model	*b*_inert_/*b*_cap_=0.3, *b*_wall_/*b*_cap_=−0.2, *b*_cap_<0	1.11	77.9°	1.06	94.8°
Squirmer, first two squirming modes only	*B*_2_/*B*_1_=0.3	1.64	102°	Below 1.02	Around 45°
Effective squirmer	*b*_inert_/*b*_cap_=−0.8, *b*_cap_<0	1.063	69.7°	1.09	65.3°

For each model, we list the parameters that give the best fit to the experimental observations. For each model and set of best-fit parameters, we give the height and orientation of the particle when it is in a ‘sliding state' above a planar wall in both the presence of gravity (corresponding to motion above a substrate) and the absence of gravity (corresponding to motion near a side wall). Experimentally, it is observed that *θ*_eq_≈90° in both cases. Of the three models, the full model shows the best fit with these experimental observations. For the squirmer with only the first two squirming modes, there are clear signs of an attractor with *h*_eq_/*R* below the numerical cutoff of *h*/*R*=1.02 in the presence of gravity, but this attractor has *θ*_eq_ far from 90° (see also Supplementary Note 3 and Supplementary Table 1). The best-fit effective squirmer agrees moderately well with the experimental observations. However, the orientation of the sliding seems significantly different from the experiment and, as discussed in Supplementary Note 3 and Supplementary Table 2, the best-fit parameters correspond to an unrealistically large force dipole.
