# Supplementary material for: Topographical pathways guide chemical microswimmers
Source: Nat Commun. 2016 Feb 9;7:10598. doi: 10.1038/ncomms10598 (PMC4748132; doi:10.1038/ncomms10598)
Supplement: Supplementary Information — Supplementary Figures 1-5, Supplementary Tables 1-2, Supplementary Notes 1-5 and Supplementary References [file ncomms10598-s1.pdf]

SUPPLEMENTARY INFORMATION

SUPPLEMENTARY FIGURES

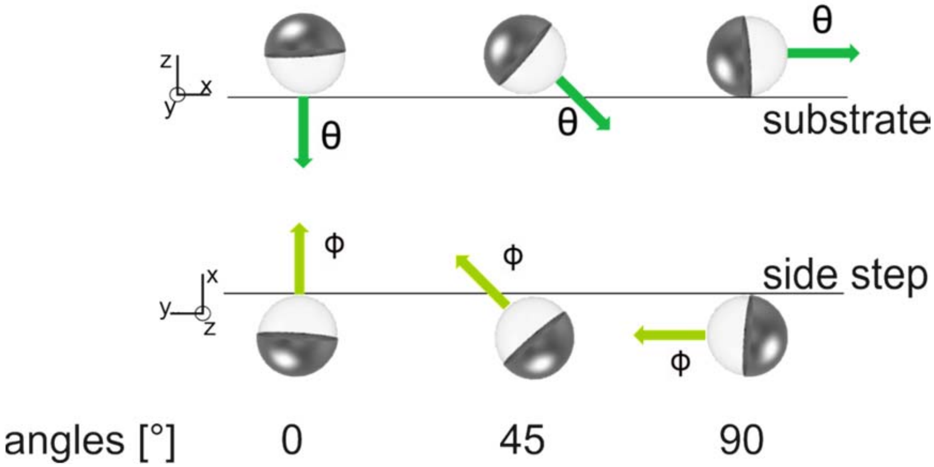

Supplementary Figure 1 | Definition of the angles of the microswimmer symmetry axis relative to the substrate and the side step.

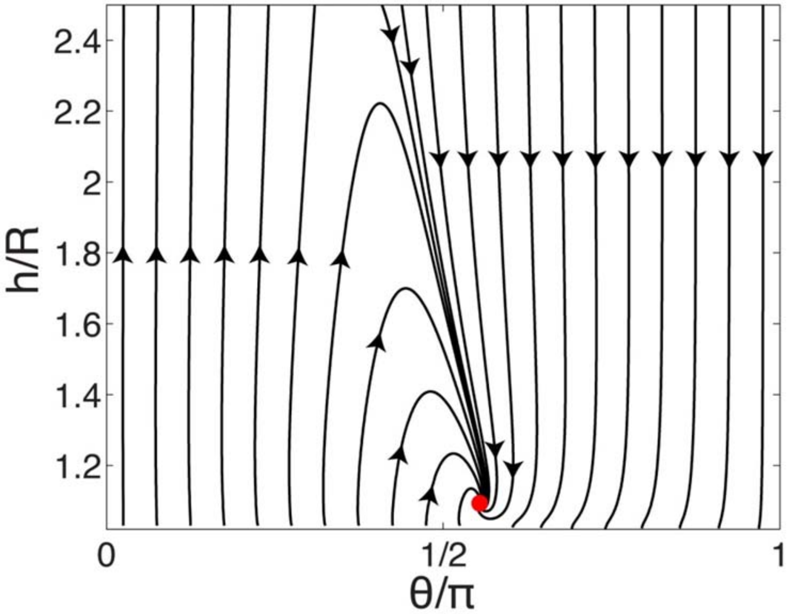

Supplementary Figure 2 | Phase portrait for orientation parallel to the substrate of a microswimmer with R = 1.0 μm, including the effect of gravity. Except for the size of the particle, all parameters are the same as in Fig. 2d in the main text.

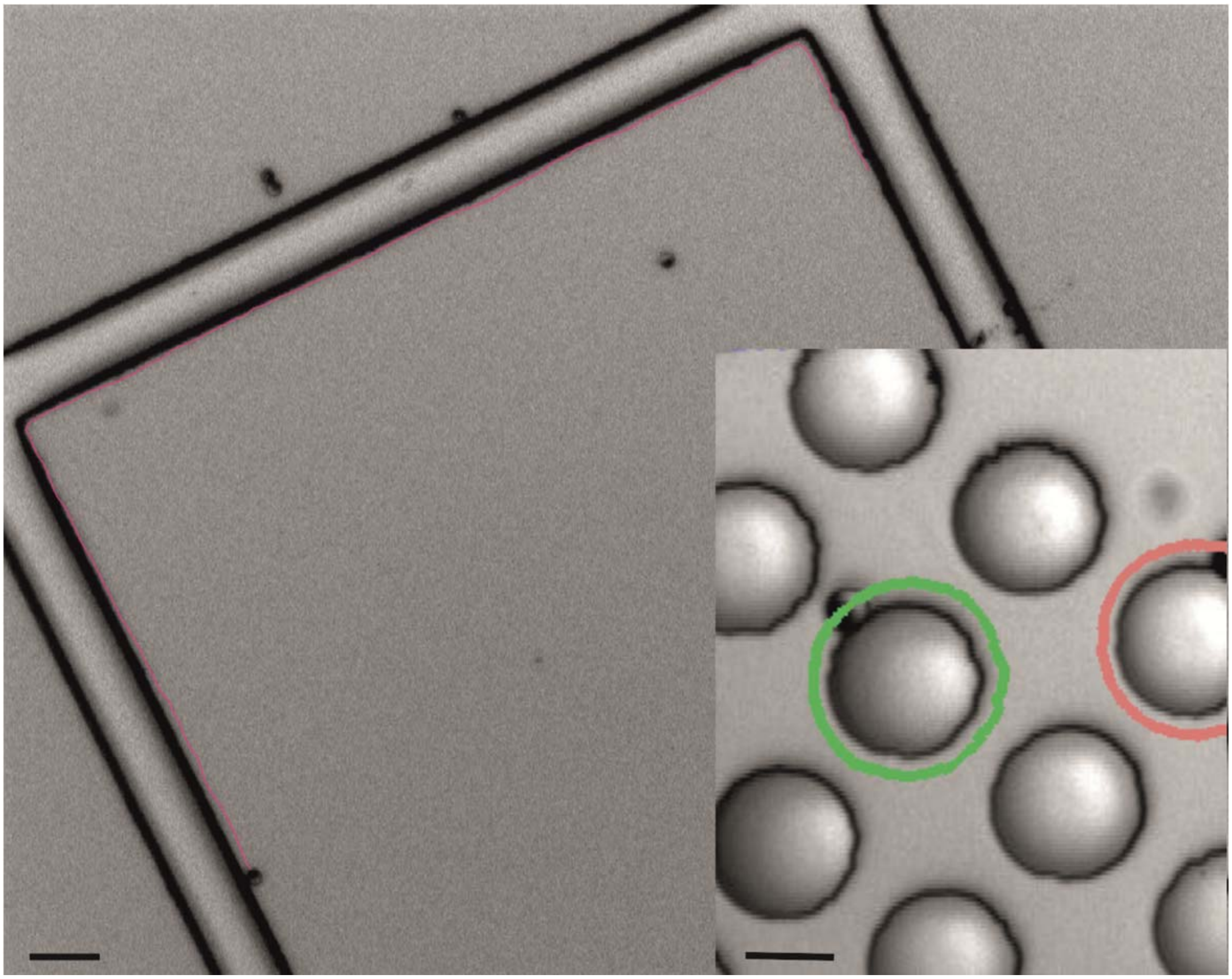

Supplementary Figure 3 | 2.5 μm Janus microswimmer trajectories following well features for 227s; scale bar corresponds to 20 μm. **Inset:** 2.5 μm Janus microswimmer circling around a step for more than 89s; both tracks were recorded in 5 vol% H_2O_2, scale bar corresponds to 10 μm.

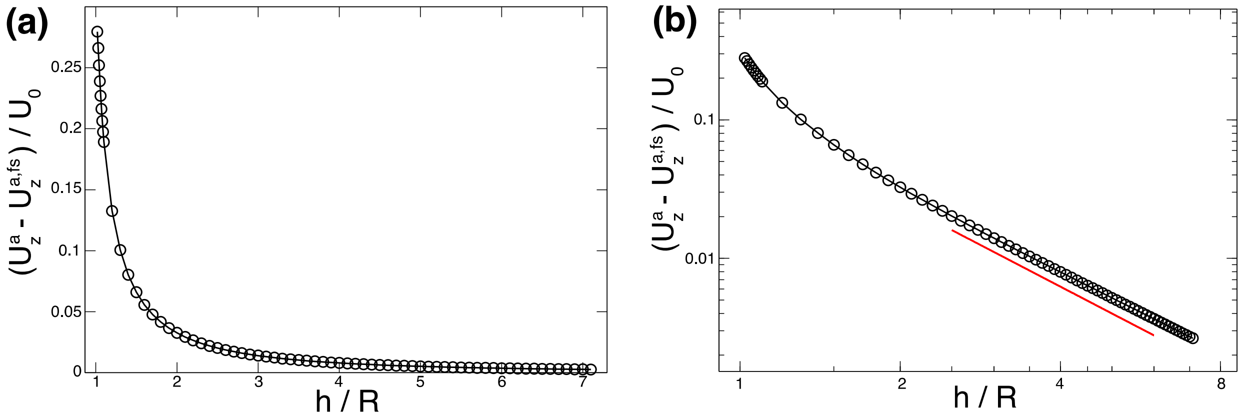

Supplementary Figure 4 | Wall-induced change of the particle velocity **(a),** for a particle with its cap oriented towards the wall ($\theta = 0°$). Chemi-osmotic and gravitational effects are not included. (**b),** Same plot as in **(a)**, but with a log-log scale. The red line shows a $(h/R)^{-2}$ scaling.

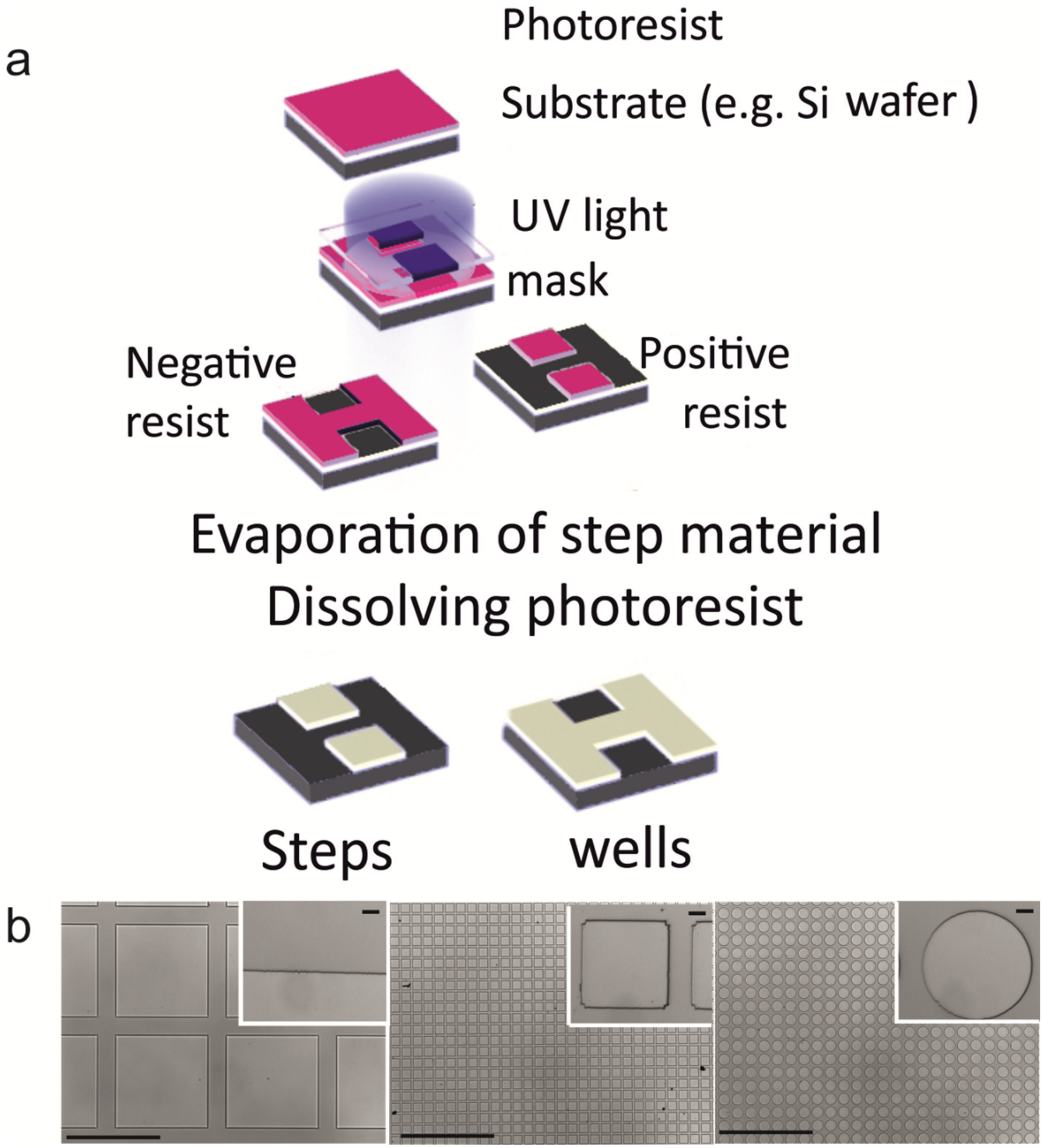

Supplementary Figure 5 | Lithography based creation of patterns **a,** Schematic representing the lithography based method to create submicron step and well structures for particle guidance. **b,** Optical images of the resulting patterns; the scale bar in the inset correspond to 10 μm, while the scale bars in the main images correspond to 500 μm.

SUPPLEMENTARY TABLES

B_2/B_1	h_{eq}/R, no gravity	θ_{eq}, no gravity	h_{eq}/R, with gravity	θ_{eq}, with gravity
-7	unstable	unstable	1.16 and 1.04 (backwards)	51.5° and 138° (backwards)
-5	unstable	unstable	1.19 and 1.03 (backwards)	49.4° and 142° (backwards)
-3	unstable	unstable	1.26	42.2°
-1	none	none	1.39	0°
0	none	none	1.24	0°
1	none	none	Below 1.02	0°
3	1.64	102°	Below 1.02	Around 45°
5	1.22	114°	Below 1.02	Around 52°
7	1.17	117°	1.032 (backwards)	53.5° (backwards)

Supplementary Table 1 | Attractor states for a squirmer with $R = 2.5\,\mu m$ and $B_l = 0$ for $l > 2$. "Unstable'' indicates that there is an unstable fixed point. "None" indicates that there is no fixed point. "Backwards" indicates that the squirmer (which in the bulk swims "away" from the cap) swims towards its cap when in the steady state near the wall, which is not observed experimentally. In some cases (indicated in the table as "Below 1.02"), there are clear signs of an attractor with h_{eq}/R below the numerical cut-off of $h/R = 1.02$; however, the corresponding orientations are significantly different from the values $\approx 90°$ observed in experiments.

β	B_1	B_2	B_3	B_2/B_1	h_{eq}/R, no gravity	θ_{eq}, no gravity	h_{eq}/R, with gravity	θ_{eq}, with gravity
-0.9	0.019	0.413	0.033	22.04	1.05, 1.03 (bwds)	68.9°, 109° (bwds)	1.056, 1.03 (bwds)	66.8°, 111° (bwds)
-0.8	0.038	0.392	0.066	10.44	1.063	69.7°	1.09	65.3°
-0.7	0.056	0.370	0.098	6.57	1.075	70.2°	1.16	59.9°
-0.6	0.075	0.348	0.131	4.64	1.094	71°	1.3	48.5°
-0.5	0.094	0.326	0.164	3.48	~1.15 (limit cycle)	~72° (limit cycle)	1.56	0°

-0.4	0.113	0.305	0.197	2.71	unstable	unstable	1.48	0°
-0.3	0.131	0.283	0.230	2.15	unstable	unstable	1.42	0°
-0.1	0.169	0.239	0.295	1.42	none	none	1.32	0°
0	0.188	0.218	0.328	1.16	none	none	1.29	0°
0.1	0.206	0.196	0.361	0.949	none	none	1.26	0°
0.3	0.244	0.152	0.427	0.625	none	none	1.21	0°
0.7	0.319	0.065	0.558	0.205	none	none	1.14	0°
1.3	0.431	-0.065	0.755	-0.151	none	none	1.09	0°
2.0	0.563	-0.218	0.984	-0.387	none	none	Below 1.02	0°

Supplementary Table 2 | Hydrodynamic properties and attractor locations for the effective **squirmer** with $R = 2.5\,\mu m$**.** "Bwds" indicates steady motion towards the cap, which is not observed experimentally. "Limit cycle" indicates one case with a sustained oscillation of small amplitude in h/R and θ. "Unstable" indicates the presence of an unstable fixed point. In one case, there are clear signs of a "hovering" attractor with h_{eq}/R below the numerical cut-off. Although we listed the amplitudes of the first three squirming modes, we note that the effective squirmer can also have $B_l \neq 0$ for $l > 3$.

Supplementary Note 1: Calculation of Gravitational Contribution to Particle Motion

The particle experiences a gravitational force from the weight of the spherical silica core and platinum cap, as well as a gravitational torque from the bottom-heaviness imparted by the platinum cap. For a given height and orientation of the particle, we use the BEM to calculate the hydrodynamic resistance tensor of the particle. We then obtain the gravitational contributions $\mathbf{U}^g$ and $\mathbf{\Omega}^g$ to the particle velocity as the product of the inverse of this tensor (i.e. the hydrodynamic mobility tensor) and the vector containing the six components of gravitational force and torque. (Our calculation of the gravitational contribution to velocity therefore includes the effect of hydrodynamic interaction with the planar wall.)

The linearity of the Stokes equation allows one to sum the separate contributions of activity and gravity to determine the complete translational and angular velocities as $\mathbf{U} = \mathbf{U}^a + \mathbf{U}^g$ and $\mathbf{\Omega} = \mathbf{\Omega}^a + \mathbf{\Omega}^g$. However, the two sets of velocities must be expressed in the same units. The contributions from particle activity are obtained in terms of $\mathrm{U_0} \stackrel{\text{def}}{=} |\mathrm{b_{cap}}|\kappa/\mathrm{D}$ and $\Omega_0 \stackrel{\text{def}}{=} \mathrm{U_0/R}$, i.e., as $\mathbf{U}^a/\mathrm{U_0}$ and $\mathbf{\Omega}^a/\Omega_0$. We estimate $\mathrm{U_0}$ and Ω_0 by taking $\mathrm{U_p} = 6\ \mu\mathrm{m\,s^{-1}}$ as a typical particle velocity. Within the neutral self-

diffusiophoretic framework, U_p/U_0 can be calculated analytically or numerically as a function of the material parameters of the particle, i.e. the extent of catalyst coverage and the spatial variation of surface mobility $b_s(\mathbf{r})$.[1,2] For instance, $U_p/U_0 = 0.25$ for half coverage and uniform surface mobility.[3] For a given set of material parameters, we can therefore calculate the characteristic velocity U_0 in dimensional units from U_p/U_0 and $U_p = 6\ \mu\text{m s}^{-1}$.

Supplementary Note 2: Isolation of contributions to particle motion

We seek to isolate and quantify the various physical contributions to the motion of a particle. We focus on the x −component of the angular velocity Ω_x. We recall that the linearity of the Stokes equations permits us to solve for *a*ctive, *g*ravitational, and *w*all *s*lip (chemi-osmotic) contributions separately and superpose them to obtain the full angular velocity: $\Omega_x = \Omega_x^a + \Omega_x^g + \Omega_x^{ws}$. To obtain the active contribution, we use the Lorentz reciprocal theorem. This theorem allows the problem of determination of $(\mathbf{U}^a, \mathbf{\Omega}^a)^T$ to be related to six "primed" problems with the same geometry but different boundary conditions. We obtain six coupled equations:

$$\mathbf{U}^a \cdot \mathbf{F}_j' + \mathbf{\Omega}^a \cdot \boldsymbol{\tau}_j' = - \int_{particle} \mathbf{v}_s \cdot \boldsymbol{\sigma}_j' \cdot \mathbf{n}\, dS, \;\; j = 1, \ldots, 6. \qquad (1)$$

Where $\mathbf{F}_j'$ and $\boldsymbol{\tau}_j'$ are the force and torque, respectively, exerted by quiescent fluid on a particle in steady translation (or rotation) with a no-slip boundary condition on its surface. The index j denotes steady translation in the $\hat{x}, \hat{y}$ or $\hat{z}$ direction for $j = 1, 2, 3$, respectively, or steady rotation in $\hat{x}, \hat{y}$ or $\hat{z}$ for $j = 4, 5, 6$. Likewise, $\boldsymbol{\sigma}_j'$ is the fluid stress tensor for the steadily translating or rotating particle. Further details concerning the derivation of Eq. 1 are provided in our previous work [4]. We recall that $\mathbf{v}_s = -b(\mathbf{r})\nabla_{||}c(\mathbf{r})$. From Eq. 1, we find the component

$$\Omega_x^a = \sum_{j=1}^{6} (\mathbf{R}^{-1})_{4j} \int_{particle} b(\mathbf{r})\nabla_{||}c(\mathbf{r}) \cdot \boldsymbol{\sigma}_j' \cdot \mathbf{n}\, dS. \qquad (2)$$

Here, the forces and torques $\mathbf{F}_j' = (\mathrm{F}_{jx}', \mathrm{F}_{jy}', \mathrm{F}_{jz}')^T$ and $\boldsymbol{\tau}_j' = (\tau_{jx}', \tau_{jy}', \tau_{jz}')^T$ have been compactly organized into a matrix

$$\boldsymbol{R} \equiv \begin{pmatrix} \mathrm{F}_{1x}' & \mathrm{F}_{1y}' & \mathrm{F}_{1z}' & \tau_{1x}' & \tau_{1y}' & \tau_{1z}' \\ \mathrm{F}_{2x}' & \mathrm{F}_{2y}' & \mathrm{F}_{2z}' & \tau_{2x}' & \tau_{2y}' & \tau_{2z}' \\ \mathrm{F}_{3x}' & \mathrm{F}_{3y}' & \mathrm{F}_{3z}' & \tau_{3x}' & \tau_{3y}' & \tau_{3z}' \\ \mathrm{F}_{4x}' & \mathrm{F}_{4y}' & \mathrm{F}_{4z}' & \tau_{4x}' & \tau_{4y}' & \tau_{4z}' \\ \mathrm{F}_{5x}' & \mathrm{F}_{5y}' & \mathrm{F}_{5z}' & \tau_{5x}' & \tau_{5y}' & \tau_{5z}' \\ \mathrm{F}_{6x}' & \mathrm{F}_{6y}' & \mathrm{F}_{6z}' & \tau_{6x}' & \tau_{6y}' & \tau_{6z}' \end{pmatrix}. \qquad (3)$$

The contribution in Eq. (2) to the angular velocity of the particle is shown in Fig. 3a of the main text.

We now write the activity-induced solute concentration $c(\mathbf{r}) = c^{fs}(\mathbf{r}) + \delta c(\mathbf{r})$ with a *f*ree *s*pace component $c^{fs}(\mathbf{r})$ and the wall correction $\delta c(\mathbf{r})$. Similarly, we write $\boldsymbol{\sigma}_j' = \boldsymbol{\sigma}_j'^{fs} + \delta\boldsymbol{\sigma}_j'$ and $\mathbf{R} = \mathbf{R}_{fs} + \delta\mathbf{R}$. Using these representations in the integral in Eq. 2, we may estimate various contributions to the particle rotation.

The free space angular velocity $\Omega_x^{a,fs} = \sum_{j=1}^{6} \left(\mathbf{R}_{fs}^{-1}\right)_{4j} \int_{particle} b(\mathbf{r})\nabla_{||}c^{fs}(\mathbf{r}) \cdot \boldsymbol{\sigma}_j'^{fs} \cdot \mathbf{n}\, dS$ is zero, due to the axial symmetry of the particle.

$\Omega_x^{a,hi} = \sum_{j=1}^{6} (\mathbf{R}^{-1})_{4j} \int_{particle} b(\mathbf{r})\nabla_{||}c^{fs}(\mathbf{r}) \cdot \delta\boldsymbol{\sigma}_j' \cdot \mathbf{n}\, dS$ gives the contribution to Ω_x^a strictly from *h*ydrodynamic *i*nteractions with the wall. It is plotted in Fig. 3b of the main text.

$\Omega_x^{a,sol} = \sum_{j=1}^{6} \left(\mathbf{R}_{fs}^{-1}\right)_{4j} \int_{particle} b(\mathbf{r})\nabla_{||}\delta c(\mathbf{r}) \cdot \boldsymbol{\sigma}_j'^{fs} \cdot \mathbf{n}\, dS$ gives the contribution to Ω_x^a, shown in Fig. 3(c) of the main text strictly from wall-induced *sol*ute modifications. In other words, $\Omega_x^{a,sol}$ represents phoretic rotation of the particle from wall-induced concentration gradients. Note that this term is non-zero only when $b_{cap} \neq b_{inert}$; in the case considered here, $b_{inert}/b_{cap} = 0.3$.

Finally, the term $\Omega_x^{a,\delta\delta} = \sum_{j=1}^{6} (\mathbf{R}^{-1})_{4j} \int_{particle} b(\mathbf{r})\nabla_{||}\delta c(\mathbf{r}) \cdot \delta\boldsymbol{\sigma}_j' \cdot \mathbf{n}\, dS$ is due to higher order coupling between the chemical and hydrodynamic effects of the wall. It is depicted in Fig. 3d of the main text. Interestingly, it is not necessarily small when the particle is close to the wall.

Now we turn to the other contributions to Ω_x. We show the contribution from gravitational torque in panel e of main text Fig.3: $\Omega_x^g = (\mathbf{R})^{-1}\mathfrak{F}^g$ where we define a generalized gravitational force $\mathfrak{F}^g = \left(0,0,F_z^g,\tau_x^g,0,0\right)^T$. This term depends on the size R of the particle; here, it is calculated for $R = 2.5\mu m$.

Likewise, in Fig. 3f of the main text, we show the contribution from wall slip, i.e., activity-induced chemio-osmotic flow along the wall: $\Omega_x^{ws} = \sum_{j=1}^{6} (\mathbf{R}^{-1})_{4j} \int_{wall} b_w(\mathbf{r})\nabla_{||}c(\mathbf{r}) \cdot \boldsymbol{\sigma}_j' \cdot \mathbf{n}\, dS$. This component is absent in the squirmer model, but it is significant in our case, where $b_w/b_{cap} = -0.2$.

Supplementary Note 3: Hydrodynamics-only models

We turn to the wider question of whether the "squirmer" model, in which the interaction with the wall is purely hydrodynamic, can reproduce the experimentally observation *for some set of parameters*. We recall that the slip velocity of an axisymmetric squirmer can be written as follows:

$$\mathbf{v}_s(\theta_p) = \sum_{l=1}^{\infty} B_l\, V_l(\cos(\theta_p)) \qquad (4)$$

where θ_p is an angle defined with respect to the axis of symmetry, $V_l(x) = \frac{2\sqrt{1-x^2}}{n(n+1)} \frac{d}{dx} P_l(x)$, $P_l(x)$ is the Legendre polynomial of order l, and B_l is the amplitude of squirming mode l.[5]

An exhaustive search through the squirming mode amplitudes B_l is beyond the scope of this work. Nevertheless, we can gain some insight by restricting our consideration to the first two squirming modes. The amplitude of the first mode is set by the free space swimming velocity $v_{f.s.} = 6\ \mu m\ s^{-1}$, since $v_{f.s.} = 2/3\ B_1$. We vary the ratio B_2/B_1, taking $B_l = 0$ for $l > 2$, and determine whether a sliding state emerges (i) in the *absence* of gravity and (ii) in the *presence* of gravity, which respectively represent (i) swimming near a side wall, and (ii) swimming above a substrate. The results are shown in Table 1 for $R = 2.5\ \mu m$. For situation (i), our results show good agreement with Gaffney and Ishimoto, [5] including the finding that sliding states emerge only for $B_2/B_1 \geq 3$.

We find that, for the parameters considered, the squirmer model cannot reproduce the experimental observation that a particle swims at $\theta \approx 90°$ in *both* situation (i) and situation (ii). Gravity shifts the sliding states that occur for $B_2/B_1 \geq 3$ to $\theta_{eq} \approx 45°$. This steady angle would be detectable experimentally as a large apparent coverage of the particles by catalyst. We conclude that, for the parameters considered in Supplementary Table 1, the effect of the force dipole (the strength of which is proportional to the amplitude B_2 of the second squirming mode) is *too weak* to balance the effect of gravity at $\theta \approx 90°$.

In constructing Supplementary Table 1, we made two simplifying and physically plausible assumptions: 1.) The contributions of the higher order modes ($l > 2$) to the disturbance velocity decay rapidly with distance from the swimmer, and hence contribute negligibly to interaction with the wall; and 2.) the ratio $B_2/B_1 \sim \mathcal{O}(1)$. We can relax both assumptions by considering an "effective squirmer" obtained within our model for a self-diffusiophoretic swimmer. The effective squirmer is obtained for a given $\beta = b_{inert}/b_{cap}$ by neglecting the effect of the wall on the concentration field of a self-diffusiophoretic particle, i.e., by using $c_{f.s.}$ as described in Supplementary Note 2. Additionally, chemi-osmotic effects are neglected. An effective squirmer could have, in principle, non-zero amplitude for all B_l. Secondly, as shown in Supplementary Table 2, the ratio $B_2/B_1 \longrightarrow \infty$ as $\beta \longrightarrow -1$ from above. This is because, for $\beta \approx -1$, one face of the particle is attracted by solute, and the other repelled by solute; furthermore, the strengths of attraction and repulsion are approximately equal. B_1 is therefore nearly zero, since it is proportional to the velocity of the particle.

Our results are in Supplementary Table 2. We find that, for the parameters studied, the effective squirmer cannot reproduce the experimental observations in both situation (i) and situation (ii). The closest match is for $\beta = -0.8$, with $\theta_{eq} \approx 70°$ in situation (i) and $\theta_{eq} \approx 65°$ in situation (ii). Achieving this particular sliding state requires a very strong force dipole interaction with the wall: $\frac{B_2}{B_1} \approx 10$. Is this physically plausible? A recent estimate of the force dipole strength for a catalytic Janus particle was provided by Brown *et al.*[6] For a $R \approx 1\ \mu m$ colloid that moves at $U \approx 15\ \mu m\ s^{-1}$ they estimate

$\alpha = 30\ \mu m^3 s^{-1}$. Non-dimensionalizing, and then using the expression from Gaffney and Ishimoto that connects B_2/B_1 with dimensionless α,[5] we find $B_2/B_1 \approx 2.5$. Hence, the $\beta = -0.8$ effective squirmer is both physically unlikely (having a very large B_2/B_1, i.e., a very large force dipole) *and* a poorer fit to experimental observations than the full model presented in our work. For more realistic values of the force dipole, hydrodynamics is *too weak* to, by itself, balance gravitational effects at $\theta \approx 90°$.

Supplementary Note 4: Range of interaction with wall

The interaction of a chemically active particle with a planar wall has a long-ranged character. However, we show that the amplitude (strength) of this interaction is very small except when the particle is close to the wall. This makes it difficult to detect its effects experimentally (at least with the equipment and techniques currently available to us)

In Supplementary Figure 4, we plot the wall-induced change of the wall normal component of the self-diffusiophoretic velocity of a particle that has its cap oriented towards the wall ($\theta = 0^\circ$). The subtraction of the free space self-diffusiophoretic velocity $U^{a,fs} = 0.1625\, U_0$ from U^a isolates the effect of the wall. (Note that chemi-osmotic and gravitational contributions are not included in the figure.) At h/R = 5, the contribution to U_z^a from the wall has already decayed to approximately three percent of the free space self-diffusiophoretic velocity. This change in speed is too small to be apparent when viewing an optical microscopy video of a particle near a step or a side wall. The effect of the wall is even weaker for orientations $\theta > 0^\circ$.

Our numerical calculations recover the long-ranged character of the interaction. In Supplementary Figure 4(b), we show the same plot as in (a), but with a log-log scale. Far away from the wall, ΔU_z^a follows a $(h/R)^{-2}$ power law, which is shown as a red line. The effect of the wall on the solute field decays as $1/r$, i.e., the leading order term representing the wall is an image point source. Since ΔU_z^a is proportional to the wall-induced concentration gradient, it decays as $1/r^2$.

Supplementary Note 5: Robustness of the sliding state

Finally, we comment on the robustness of the sliding states against thermal fluctuations, which were not included in our model. To this end we perform a standard linear stability analysis of our dynamical system, which may be written as $\{\dot{h} = f_1(h,\theta), \dot{\theta} = f_2(h,\theta)\}$, at a fixed point (h_{eq}, θ_{eq}) at which $\{f_1(h_{eq},\theta_{eq}) = 0,\ f_2(h_{eq},\theta_{eq}) = 0\}$. This amounts to determination of the eigenvalues $\lambda_{1,2}$ of the Jacobian matrix J evaluated at (h_{eq}, θ_{eq}), i.e.

$$J = \begin{pmatrix} \partial f_1/\partial h & \partial f_1/\partial \theta \\ \partial f_2/\partial h & \partial f_2/\partial \theta \end{pmatrix}\Bigg|_{(h_{eq},\theta_{eq})} . \qquad (5)$$

Thus, for the fixed point at Fig. 2d, we obtain that the Jacobian has eigenvalues $\lambda_{1,2} \cong (-0.40 \pm 0.17i)U_0/R$. Since the real part of the eigenvalues is negative, the fixed point is a stable attractor: a small perturbation away from the fixed point will exponentially decay with a characteristic timescale $\tau = [Re(\lambda_1)]^{-1}$. To convert this timescale into dimensional units, we use $R = 2.5\,\mu m$ and $U_0 \approx 6.2\,U_{f.s.} = 6.2 \times 6\mu m\, s^{-1}$, where $U_{f.s.}$ is the self-propulsion velocity of a half-covered Janus swimmer in a *f*ree *s*pace with $b_{inert}/b_{cap} = 0.3$.[7] We obtain the timescale $\tau \approx 0.37\,s$ for the self-trapping of a particle into this sliding state. For comparison, the characteristic timescales of rotational and translational diffusion are $\tau_r \cong 97s$ and $\tau_t \cong 70s$, respectively. This separation of timescales indicates that the sliding state in Fig. 2d is robust against thermal noise. Similarly, for the fixed points in Fig. 2e and Supplementary Fig. 3, we obtain characteristic self-trapping timescales $\tau \cong 1.7\,s$ and $\tau \cong 0.4\,s$, respectively (in the latter case we use $R = 1\,\mu m$ for the dimensionalization).

Supplementary references

(1) Golestanian, R.: Anomalous Diffusion of Symmetric and Asymmetric Active Colloids. *Phys. Rev. Lett.*, **102,** 188305, (2009)

(2) Golestanian, R.; Liverpool, T. B.; Ajdari, A.: Designing phoretic micro- and nano-swimmers, *New Journal of Physics*, **9**, 126, (2007).

(3) Popescu, M. N.; Dietrich, S.; Oshanin, G.: Confinement effects on diffusiophoretic self-propellers, *J. Chem. Phys.* **130**, 194702 (2009).

(4) Uspal, W. E., Popescu, M. N., Dietrich, S. & Tasinkevych, M. Self-propulsion of a catalytically active particle near a planar wall: from reflection to sliding and hovering. *Soft Matter*, **11**, 434-438, (2015).

(5) Ishimoto, K.; Gaffney, E. A.: Squirmer dynamics near a boundary. *Phys.Rev. E* **88,** 062702, (2013).

(6) Brown, A. T. et al.: Swimming in a Crystal. Soft Matter, Advance article (2015).

(7) Golestanian, R., Liverpool, T. B. & Ajdari, A. Propulsion of a Molecular Machine by Asymmetric Distribution of Reaction Products. *Phys. Rev. Lett*. **94**, 220801 (2005).
